# Home and social role as factors leading to health maintenance among the elderly living in rural Japan: A longitudinal study

**DOI:** 10.1016/j.heliyon.2023.e21763

**Published:** 2023-10-30

**Authors:** Shota Kuroiwa, Keiichiro Kita, Maiko Kuroiwa, Shinji Minami, Seiji Yamashiro

**Affiliations:** a Department of General Medicine, Toyama University Hospital, Japan; b Executive Policy Adviser to Nanto-city, Japan

**Keywords:** Home and social role, Elderly, Health maintenance factors, Taking care of others, Social activities, Logistic regression analysis

## Abstract

**Purpose:**

Our study aimed to clarify home and social factors by gender that lead to maintenance of health in the elderly, such as taking care of others and having social activities.

**Methods:**

A total of 14,712 and 14,799 respondents to the “Survey of Needs in the Spheres of Daily Life” conducted in Nanto City, Toyama Prefecture in 2017 and 2020, respectively, who were aged 65 years or older (recovery rate was 78.5 %) were enrolled. Of these, 4,322 people who answered that they did not receive long-term care in 2017 survey and who also responded to the 2020 survey or were confirmed dead by the time of the survey were included in the analysis. The status of health maintenance was the outcome and those who answered the 2020 survey saying they did not receive long-term care were defined as health maintained. Those who answered that they did receive long-term and those who died were defined as health lost.

**Results:**

After adjusting for variables such as basic attributes, health status, and functional capacity, the elderly who had persons whom to they provided care (excluding long-term care) had health maintenance rate higher at 3 years than those who did not provide care or long-term care. In addition, the results showed that men who had job with income and women who participated in neighborhood associations had higher rates of health maintenance.

**Conclusion:**

This study showed that older adults who take on roles at home and in society are more likely to maintain their health.

## 1 Introduction

It is important for the elderly to be able to maintain an independent life that does not require long-term care or assistance. The 2019 Healthy Life Expectancy Extension Plan in Japan set a goal of increasing healthy life expectancy by 3 years for men and women by 2040 [1]. Healthy life expectancy is considered to be influenced not only by individual health-related habits, but also by the social and home environment. Therefore, identifying these determinants is important [2]. In addition, social and home environments change in part due to the awareness and actions of elderly individuals themselves, such as participation in social activities and active care for their spouse, children, and grandchildren.

Studies have shown that the active engagement of elders with their surroundings is associated with both health maintenance and longevity. For example, quantitative empirical studies have shown that positive social activities (social relationships) tend to reduce the risk of death [[3], [4], [5]], disability [6], and dementia [7], and reduce care needs [[8], [9], [10]]. Researchers have speculated that this might be due to the socio-psychological benefits of social participation among the elderly, such as improving self-esteem, creating a sense of purpose in life, and relieving stress [11,12]. In addition, social participation can help elderly individuals avoid loneliness, which has a negative impact on their physical and mental health, mortality, and their sense of well-being [13].

In contrast, some studies have shown that elderly individuals’ provision of personal support to family members (such as spouse, children, and grandchildren) and friends affects their own health [[14], [15], [16]]; however, the number of studies on this topic is inadequate. In particular, there is a need to understand the impact of the provision of long-term care by the elderly on the maintenance of their own health.

Based on this awareness of the problem, the purpose of this study is to comprehensively identify the household and social factors that contribute to the maintenance of health among the elderly based on sex. In particular, we focus on matters in which elderly individuals themselves can be actively involved and clarify the influence of caring for others on their own health maintenance as well as the influence of each specific social activity on the elderly's own health maintenance. In addition, we will identify the rates of implementation and participation in these household care and social activities and the characteristics by gender.

## 2 Methods

This study used data from the “Survey of Needs in the Spheres of Daily life” conducted by mail or visit to all elderly people aged 65 years and older in Nanto, Toyama, Japan, from June to August 2017 and June to August 2020. This survey is conducted every 3 years in many local governments. Our study was a longitudinal study between two time points, with the 2017 survey results considered as the baseline and the health maintenance status as of the 2020 survey as the outcome.

Data were constructed by matching response data and transfer (death) information between the two surveys. The surveys were completed by 14,712 of 18,753 respondents in 2017 and 14,799 of 18,845 respondents in 2020 (a combined response rate of 78.5 %).

First, we selected 6,085 respondents from the 2017 survey with no missing values in the explanatory variable data used in the analysis, based on survey nonresponse and other factors. Next, to align the eligibility criteria, we defined those who responded in the 2017 survey that were not receiving nursing care or assistance as “health maintainers” and extracted them for analysis. As a result, we extracted 5,078 individuals.

**Fig. 1:**
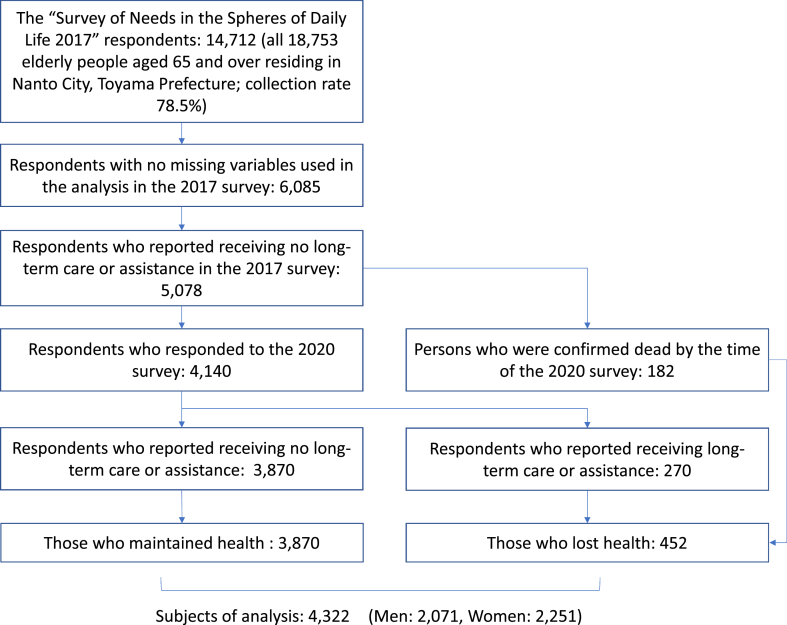
Flowchart of subject extraction analysis.

Of these individuals, a total of 4,322 persons (2,071 men and 2,251 women) were further selected for analysis, including 4,140 who responded to the 2020 survey and 182 whose death was confirmed by the time of the 2020 survey (Fig. 1).

Thus, the target population for this study was older adults aged ≥65 years who were not receiving long-term care or assistance at baseline and who have maintained a healthy life expectancy.

### 2.1 Analysis of items

Multiple logistic regression analysis was conducted with health maintenance or loss at the time of the 2020 survey as the objective variable and basic attributes, health, living situation, and other factors at baseline were considered explanatory variables.

Similar to the definition at the baseline, those who answered that they did not receive long-term care in the 2020 survey were considered healthy, and those who answered that they did receive long-term care and those who died were defined as health lost. In Japan, “the period during which activities of daily living are independent” can be used as an index for calculating a healthy life expectancy. In this paper, due to data restrictions, the state of not receiving long-term care or assistance in daily life was defined as a state of maintaining good health [2].

Explanatory variables included basic attributes at baseline, health and life functions, lifestyle, and economic status indicators, as well as providing long-term care and care, participation in various social activities, and their subjective sense of well-being.

Regarding the selection of variables, we obtained as broad a range as possible from the data of the “Survey of Needs in the Spheres of Daily life” while referring to existing studies dealing with the relationship between the home and social environments and health maintenance among the elderly [[8], [9], [10], [11],^17^].

We used the following steps to create categories for the provision of long-term care or care by the subject. First, we ascertained whether the subject had partners to whom they provided long-term care or care. This definition of “care” is broad and includes meal preparation, shopping, transportation, etc. for spouses, children, and others. Next, subjects who had partners to whom they provided long-term care or care were categorized according to whether or not that partner was in need of long-term care.

In previous studies, it was pointed out that Japanese people are more likely to be reluctant to rely on people other than their families for nursing care [18,19], so long-term care and care were generally considered as intra-family activities.

Regarding the status of participation in various social activities, we considered whether the person participated in a job with income, neighborhood associations, senior citizens' clubs, hobby-related groups, volunteer groups, and sports-related groups. For each social activity, participants were defined as those who indicated that they participated in the activity at least several times a year.

For subjective well-being, we employed an 11-point question in which respondents answered the degree of their well-being on a scale of 0–10. The distribution of responses were defined as 0 to 5 indicating “not happy,” 6 to 8 indicating “a little happy,” and 9 to 10 indicating “happy.”

Subjective well-being in the elderly is important in its own right; moreover, it has attracted attention as a factor that influences survival prognosis [20,21]. Some reports suggest that even a simple self-assessment of subjective well-being using four options can be a predictive indicator of 3-year survival [22]. Therefore, in this study, we also examined the effect of subjective well-being on health maintenance among the elderly.

Age and family structure were used as basic attributes. For age, six categories were used, ranging from “65–69 years” at baseline to “90+ years” every 5 years. Family composition was divided into four groups: “one-person household,” “two-person household with a married couple,” “two-household with parents and children,” and “other.”

For health and living functions, we considered the body mass index (BMI), subjective health, the instrumental activity of daily living index (IADL), and the intellectual activity index in the Tokyo Metropolitan Institute of Gerontology Index of Competence [23,24].

Regarding BMI, based on the diagnostic criteria for obesity [25], the degree of obesity was categorized into three groups. For subjective health, the answers to the four-point scale question answers were used as is.

For smoking and drinking, we used yes-no answers to the questions asking if they smoked or not. For the economic situation, the answers to the five-step questions were used as they were.

### 2.2 Analytical method

First, in order to ascertain the actual living conditions of the respondents, the frequency and composition ratio of the objective variable used for logistic regression analysis and each category for each explanatory variable were obtained for each gender, and the difference between the genders was analyzed. In addition, we also obtained the specific person to whom the subject provided long-term care or care.

Finally, a binomial logistic regression analysis was conducted to determine the rate of health maintenance for each explanatory variable by gender and to examine whether providing long-term care or care, participating in various social activities, and having a high subjective sense of well-being were associated with health maintenance at 3 years, even after adjusting for various other variables.

When the variable inflation factor of each variable was confirmed during the analysis, ranging between 1.04 and 4.89 in men and 1.05 and 5.04 in women, and all variables were less than 10.00, it was judged that there was no problem with multicollinearity.

All statistical analyses were performed using IBM SPSS Statistics version 26.

## 3 Results

Three years after the baseline analysis, the overall health maintenance rate was 89.5 % for all participants, 90.1 % for men, and 89.0 % for women, with no significant difference between men and women.

Regarding the presence or absence of providing long-term care or care, 30.4 % of the respondents answered, “providing care to no one,” 44.3 % answered “providing care,” and 25.2 % answered “providing long-term care.“

Significant differences were found in the composition ratio of the three groups between men and women, and there was a tendency for men to have a higher ratio of “providing care” and for women to have a high ratio of “providing care to no one” and “providing long-term care.”

In terms of social participation, the overall participation rate in each activity was 42.1 % for work with income, 58.9 % for participated in neighborhood associations, 51.8 % for participated in elderly clubs, and 37.7 % for participated in hobby-related groups, 28.7 % participated in a volunteer group, and 28.4 % participated in sports-related groups.

The ratio of men who participated was significantly higher in the work with income, neighborhood associations, elderly clubs, and sports-related groups categories, and the ratio of women who participated was significantly higher in the hobbies-related group. There was no significant difference between the ratio of men and women in the volunteer group.

The overall composition of subjective happiness was 18.9 % “happy,” 48.8 % “somewhat happy,” and 32.3 % “not happy,” with a significantly higher tendency among women.

Other significant differences between men and women regarding the explanatory variables used in the logistic regression analysis were in age, family structure, BMI, IADL, intellectual activity, smoking, alcohol, and economic situation. There was no significant difference in subjective health between the men and women (Table 1).

**Table 1:** Demographics.

		All (n = 4,322)		Men (n = 2,071)		Women (n = 2,251)		P value^a^
		n (%)		n (%)		n (%)		
maintained good health after 3 years								
	Maintain	3,870	(89.5)	1867	(90.1)	2003	(89.0)	P = 0.210
	lost	452	(10.5)	204	(9.9)	248	(11.0)	
providing long-term care or care								
	providing care to no one	1,315	(30.4)	609	(29.4)	706	(31.4)	P = 0.031
	providing long-term care	1,091	(25.2)	501	(24.2)	590	(26.2)	
	providing care	1,916	(44.3)	961	(46.4)	955	(42.4)	
received income for work								
	No	2,502	(57.9)	1010	(48.8)	1492	(66.3)	P < 0.000
	yes	1,820	(42.1)	1061	(51.2)	759	(33.7)	
neighborhood associations								
	no participation	1,778	(41.1)	596	(28.8)	1182	(52.5)	P < 0.000
	participation	2,544	(58.9)	1475	(71.2)	1069	(47.5)	
elderly club								
	no participation	2,085	(48.2)	935	(45.1)	1150	(51.1)	P < 0.000
	participation	2,237	(51.8)	1136	(54.9)	1101	(48.9)	
hobby-related group								
	no participation	2,692	(62.3)	1364	(65.9)	1328	(59.0)	P < 0.000
	participation	1,630	(37.7)	707	(34.1)	923	(41.0)	
volunteer group								
	no participation	3,080	(71.3)	1483	(71.6)	1597	(70.9)	P = 0.631
	participation	1,242	(28.7)	588	(28.4)	654	(29.1)	
sports-related group								
	no participation	3,094	(71.6)	1400	(67.6)	1694	(75.3)	P < 0.000
	participation	1,228	(28.4)	671	(32.4)	557	(24.7)	
subjective well-being								
	not happy	1,395	(32.3)	735	(35.5)	660	(29.3)	P < 0.000
	slightly happy	2,109	(48.8)	1001	(48.3)	1108	(49.2)	
	happy	818	(18.9)	335	(16.2)	483	(21.5)	
age								
	65–69 years old	1,798	(41.6)	917	(44.3)	881	(39.1)	P < 0.000
	70–74 years old	1,034	(23.9)	492	(23.8)	542	(24.1)	
	75–79 years old	711	(16.5)	346	(16.7)	365	(16.2)	
	80–84 years old	447	(10.3)	193	(9.3)	254	(11.3)	
	85–89 years old	242	(5.6)	93	(4.5)	149	(6.6)	
	90 years old and over	90	(2.1)	30	(1.4)	60	(2.7)	
family structure								
	Alone	386	(8.9)	130	(6.3)	256	(11.4)	P < 0.000
	living with a couple	1,420	(32.9)	811	(39.2)	609	(27.1)	
	Two households	1,312	(30.4)	573	(27.7)	739	(32.8)	
	Other	1,204	(27.9)	557	(26.9)	647	(28.7)	
BMI								
	Normal (between 18.5 and 25.0)	3,047	(70.5)	1462	(70.6)	1585	(70.4)	P < 0.000
	Low (18.5 and less)	328	(7.6)	102	(4.9)	226	(10.0)	
	High (25.0 and above)	947	(21.9)	507	(24.5)	440	(19.5)	
subjective health								
	very good	351	(8.1)	183	(8.8)	168	(7.5)	P = 0.472
	good	3,013	(69.7)	1406	(67.9)	1607	(71.4)	
	not good	832	(19.3)	412	(19.9)	420	(18.7)	
	bad	126	(2.9)	70	(3.4)	56	(2.5)	
IADL								
	high (5 points)	3,734	(86.4)	1697	(81.9)	2037	(90.5)	P < 0.000
	slightly low (4 points)	383	(8.9)	293	(14.1)	90	(4.0)	
	low (3 points or less)	205	(4.7)	81	(3.9)	124	(5.5)	
intellectual activity								
	high (4 points)	2,639	(61.1)	1199	(57.9)	1440	(64.0)	P < 0.000
	slightly low (3 points)	1,120	(25.9)	590	(28.5)	530	(23.5)	
	low (2 points or less)	563	(13.0)	282	(13.6)	281	(12.5)	
smoking								
	no	3,886	(89.9)	1675	(80.9)	2211	(98.2)	P < 0.000
	yes	436	(10.1)	396	(19.1)	40	(1.8)	
alcohol consumption								
	no	2,501	(57.9)	694	(33.5)	1807	(80.3)	P < 0.000
	yes	1,821	(42.1)	1377	(66.5)	444	(19.7)	
economic situation								
	very difficult	279	(6.5)	165	(8.0)	114	(5.1)	P = 0.003
	slightly difficult	943	(21.8)	473	(22.8)	470	(20.9)	
	normal	2,735	(63.3)	1250	(60.4)	1485	(66.0)	
	Comfortable	329	(7.6)	169	(8.2)	160	(7.1)	
	very comfortable	36	(0.8)	14	(0.7)	22	(1.0)	

a A P value is the test result for a comparison between men and women. A χ2 test was used for providing care, family structure, smoking, drinking, and health maintenance status after 3 years. Other variables were analyzed with a Wilcoxon's rank sum test.

When the respondents tabulated the partners (multiple responses) for whom they provided long-term care or care, 55.2 % were “spouse,” 18.0 % were “relatives, parents, grandchildren, brothers, and sisters,” 13.7 % were “children living together,” and 9.2 % were “children living separately,” 1.4 % were “friends,” and 0.7 % were “neighbors.” Of these, men were significantly higher in the “spouse” category and women were significantly higher in “relatives, parents, grandchildren, siblings,” “children living together,” “children living separately,” and “friends” categories (Table 2).

**Table 2:** Persons who received long-term care or care (multiple responses).

	All (n = 4,322)		Men (n = 2,071)		Women (n = 2,251)		P value
	n (%)		n (%)		n (%)		
persons who received long-time care or care (multiple responses)							
spouse	2,387	(55.2)	1,270	(61.3)	1,117	(49.6)	P < 0.000
relatives, parents, grandchildren, brothers, and sisters	777	(18.0)	286	(13.8)	491	(21.8)	P < 0.000
children living together	593	(13.7)	186	(9.0)	407	(18.1)	P < 0.000
children living separately	398	(9.2)	129	(6.2)	269	(12.0)	P < 0.000
friends	62	(1.4)	18	(0.9)	44	(2.0)	P = 0.003
neighborhood	32	(0.7)	13	(0.6)	19	(0.8)	P = 0.407
other	78	(1.8)	30	(1.4)	48	(2.1)	P = 0.092

A P value is the test result for a comparison between men and women. A χ2 test was used for other variables.

Next, we confirmed the odds ratio for each category of explanatory variables, which was the result of logistic regression analyses.

First, regarding “providing long-term care or care,” for both men and women, the odds ratio was significantly higher for “providing care” than for “providing care to no one.” However, there was no significant difference between “providing long-term care” and “providing care to no one.”

Subsequently, regarding the status of participation in various social activities, the odds ratios were significantly higher for men who participated in work with income and for women who participated in neighborhood associations.

In contrast, in terms of subjective well-being, there was no significant difference between the men and women groups.

Other explanatory variables showing significant differences for both men and women were age and IADL. BMI for men only, and family structure and subjective health and alcohol for women only showed significant differences (Table 3).

**Table 3:** Relationship between subject status and health maintenance rate after 3 years and odds ratio by multiple logistic regression analysis.

		Men (n = 2,071)				Women (n = 2,251)			
variables	category	health maintenance ratio (%)	odds ratio (95 % confidence interval)		P value	health maintenance ratio (%)	odds ratio (95 % confidence interval)		P value
providing long-term care or care									
	providing care to no one	84.7	1	(reference)		78.9	1	(reference)	
	providing long-term care	94.3	1.03	(0.67–1.56)	P = 0.907	92.4	1.25	(0.80–1.97)	P = 0.326
	providing care	88.8	1.57	(1.03–2.37)	P = 0.034	94.3	1.55	(1.02–2.35)	P = 0.041
received income for work									
	no participation	84.6	1	(reference)		85.2	1	(reference)	
	participation	95.5	1.57	(1.06–2.34)	P = 0.025	96.4	1.16	(0.71–1.89)	P = 0.551
neighborhood association									
	no participation	82.2	1	(reference)		83.2	1	(reference)	
	participation	93.4	0.93	(0.62–1.40)	P = 0.725	95.4	1.73	(1.14–2.62)	P = 0.009
elderly club									
	no participation	86.7	1	(reference)		85.6	1	(reference)	
	participation	93.0	1.26	(0.86–1.85)	P = 0.232	92.6	1.13	(0.77–1.65)	P = 0.528
hobby-related groups									
	no participation	88.3	1	(reference)		84.9	1	(reference)	
	participation	93.6	1.05	(0.69–1.61)	P = 0.814	94.8	1.04	(0.68–1.59)	P = 0.856
volunteer group									
	no participation	88.5	1	(reference)		86.3	1	(reference)	
	participation	94.2	0.90	(0.57–1.43)	P = 0.664	95.4	0.90	(0.54–1.47)	P = 0.665
sports-related groups									
	no participation	87.9	1	(reference)		86.5	1	(reference)	
	participation	94.8	1.30	(0.83–2.04)	P = 0.259	96.4	1.40	(0.81–2.41)	P = 0.223
subjective well-being									
	happy	91.3	1	(reference)		90.5	1	(reference)	
	slightly happy	92.7	1.25	(0.75–2.09)	P = 0.391	91.3	1.25	(0.80–1.96)	P = 0.332
	not happy	86.1	0.87	(0.52–1.47)	P = 0.613	83.9	0.69	(0.42–1.12)	P = 0.131
age									
	65–69 years old	95.9	1	(reference)		97.4	1	(reference)	
	70–74 years old	93.5	0.57	(0.34–0.95)	P = 0.032	95.4	0.62	(0.34–1.14)	P = 0.125
	75–79 years old	87.9	0.34	(0.20–0.57)	P < 0.000	92.1	0.41	(0.22–0.75)	P = 0.004
	80–84 years old	75.1	0.22	(0.13–0.37)	P < 0.000	76.8	0.17	(0.09–0.30)	P < 0.000
	85–89 years old	67.7	0.18	(0.09–0.34)	P < 0.000	50.3	0.06	(0.03–0.12)	P < 0.000
	90 years old and over	53.3	0.09	(0.04–0.24)	P < 0.000	36.7	0.05	(0.02–0.11)	P < 0.000
family structure									
	alone	84.6	1	(reference)		83.6	1	(reference)	
	living with a couple	90.6	1.35	(0.71–2.57)	P = 0.358	92.8	1.01	(0.57–1.79)	P = 0.978
	two house holds	88.7	1.40	(0.72–2.70)	P = 0.319	86.9	1.37	(0.83–2.27)	P = 0.221
	other	92.3	1.58	(0.80–3.11)	P = 0.187	90.0	1.77	(1.03–3.06)	P = 0.040
BMI									
	Normal (between 18.5 and 25.0)	89.2	1	(reference)		89.3	1	(reference)	
	low (18.5 or less)	82.4	1.16	(0.61–2.21)	P = 0.647	83.2	1.02	(0.62–1.67)	P = 0.941
	high (25.0 or over)	94.5	2.07	(1.31–3.27)	P = 0.002	90.7	1.11	(0.73–1.71)	P = 0.622
subjective health									
	very good	93.4	1	(reference)		94.0	1	(reference)	
	slightly good	93.0	1.39	(0.71–2.74)	P = 0.342	91.7	0.81	(0.37–1.78)	P = 0.594
	not good	82.5	0.66	(0.32–1.36)	P = 0.259	79.5	0.55	(0.24–1.27)	P = 0.160
	bad	68.6	0.48	(0.19–1.21)	P = 0.120	67.9	0.33	(0.11–0.93)	P = 0.036
IADL									
	high (5 points)	92.7	1	(reference)		92.9	1	(reference)	
	slightly low (4 points)	87.4	0.82	(0.52–1.29)	P = 0.391	70.0	0.94	(0.52–1.70)	P = 0.846
	low (3 points and less)	46.9	0.21	(0.11–0.41)	P < 0.000	37.9	0.34	(0.20–0.58)	P < 0.000
Intellectual activity									
	high (4 points)	93.1	1	(reference)		94.1	1	(reference)	
	slightly low (3 points)	89.8	0.77	(0.52–1.13)	P = 0.178	85.1	0.84	(0.57–1.25)	P = 0.398
	low (2 points and less)	78.4	0.63	(0.39–1.03)	P = 0.068	70.1	0.75	(0.47–1.19)	P = 0.226
smoking									
	no	89.7	1	(reference)		89.0	1	(reference)	
	yes	91.9	0.921	(0.59–1.44)	P = 0.720	87.5	0.51	(0.16–1.62)	P = 0.255
alcohol consumption									
	no	85.7	1	(reference)		87.2	1	(reference)	
	yes	92.4	1.064	(0.76–1.50)	P = 0.724	96.2	1.88	(1.08–3.28)	P = 0.026
economic situation									
	very difficult	87.3	1	(reference)		93.9	1	(reference)	
	slightly difficult	91.5	1.36	(0.72–2.60)	P = 0.345	89.1	0.35	(0.13–0.91)	P = 0.031
	normal	89.3	0.93	(0.52–1.68)	P = 0.815	88.6	0.33	(0.13–0.83)	P = 0.018
	slightly comfortable	95.3	1.39	(0.54–3.57)	P = 0.492	88.1	0.31	(0.10–0.92)	P = 0.036
	very comfortable	92.9	0.79	(0.07–8.48)	P = 0.848	90.9	2.01	(0.24–16.58)	P = 0.516
model χ2 test			P = 0.000				P = 0.000		
Hosmer-Lemeshow test			χ^2^ = 4.78 df = 8 P = 0.781				χ^2^ = 9.45 df = 8 P = 0.306		
percentage of correct classifications			91.3 %				90.9 %		

Objective variables: health maintenance = 1, health loss = 0.

## 4 Discussion

The results of this study show that even after adjusting for other factors, “care-providing” elderly adults have better health maintenance at 3 years in both men and women compared with “non–care-providing” elderly adults. As for social participation, there was a positive relationship between participation in “work with income” for men and participation in “neighborhood associations” for women with health maintenance after 3 years. In contrast, there were no significant differences in health maintenance after 3 years related to other forms of social participation.

From this, it can be inferred that living with responsibilities and roles, regardless of whether in a home or a social environment, leads to the maintenance of health for both men and women.

For example, having persons to care for, whether it is a spouse or a son, means that one has a responsibility to that partner. In addition, having a job that provides an income and participating in neighborhood associations can lead to assuming certain responsibilities and having a role in the workplace and community.

However, the provision of long-term care is burdensome and does not always lead to health maintenance. In addition, participation in elderly clubs, hobbies, and sports-related groups is primarily for their own sake and entertainment, and is weak in terms of developing a sense of responsibility and role within a group. Volunteering can be said to be for the benefit of others, but in terms of a sense of obligation, it tends not to be as strong as work in the workplace or in neighborhood associations. This result is consistent with previous studies that reported being in a leadership position [11] or participating in more practice-oriented activities [26] tended to lower the mortality rate compared to simply participating in social activities. Therefore, it was inferred that an appropriate workload, plus a sense of obligation to others and a role with responsibility, is particularly important for the maintenance of health among the elderly.

Based on the above, we believe that this study is unique in that it expands on the findings of previous studies suggesting there is a positive relationship between the strength of social relationships and health or mortality prevention [4,5,[27], [28], [29], [30]], or between caregiving for others and volunteer activities by the elderly and quality of life (QOL) [31,32]. This comprehensively shows that having roles to care for others at home and in society helps elderly people maintain health.

The results of this survey showed that 69.6 % of the respondents had a partner to whom they provide long-term care or care. In contrast, the percentages of providing long-term care or care to neighbors and friends were only 0.7 % and 1.4 %, respectively, and in many cases, long-term care or care is directed to spouses and children living together or separated. Therefore, in this study, as well as the previous studies in Japan [[17], [18], [19]], the provision of long-term care and care by the elderly is generally a domestic and private act within families rather than society at large.

Currently, the number of elderly people living alone in rural areas of Japan is increasing significantly due to the rapid aging of the population and the declining birthrate. In such cases, the fact that the provision of long-term care and care is confined to relatives is one of the major challenges in terms of local medical care and welfare.

If the findings of this study indicating that care providers have positive health benefits become more generalized, this could be one basis for encouraging older adults to help each other inside and outside the family.

In this sense, the results of this study also shed light on the kind of advice that medical and welfare institutions and local governments should provide to the elderly so that they can maintain a healthy life expectancy.

In addition to factors that contribute to the maintenance of healthy life expectancy, future research should also identify health and habits that contribute to the maintenance and improvement of well-being and QOL, as well as family and social factors. This is because we believe that medical services should contribute not only to the maintenance of health but also to the maintenance and improvement of well-being and QOL of the elderly. Therefore, we would like to promote interdisciplinary research that goes beyond the existing framework of medicine, including the use of findings from sociology and social psychology.

Finally, the following limitations should be mentioned.

The first is the limitation of the survey data used in this study. The “Survey of Needs in the Spheres of Daily Life” was conducted by many municipalities based on the guidance of the Ministry of Health, Labor and Welfare, but most of them were sample surveys, and those who need long-term care were excluded from the survey. In contrast, Nanto City, the target area of this study, conducts a survey of the entire population, and can be said to be very proactive in its efforts.

In contrast, the elderly are inevitably burdened with answering the survey, resulting in a large number of unknown values. In particular, when data from two time points are matched and many variables are attempted to be utilized in the analysis, as in this study, the proportion of missing cases tends to be high, which may result in a selection bias. Although there are limitations because this is a survey of the elderly, we should improve the accuracy of the responses by making the survey form easier to understand and by providing a process to reconfirm unknown values with the subjects.

Second, there is the limitation of the research target. To align the target population conditions for this study, only older adults who were in good health as of 2017 were included. Therefore, we were not able to clarify the effects of taking care of others and participating in society for the elderly who had already lost their health. We would like to clarify this point in the future.

Finally, there is a limitation in the generalizability of the study results. Although this study is the result of an analysis of a large-scale survey with continuity, it was conducted in one rural area, and the results may differ from those obtained in other areas, such as urban areas. Further generalization of the study results, such as validation of cross-validity, is needed.

## 5 Conclusion

This study aimed to explore both home and social factors that lead to the maintenance of the health of the elderly. As a result, even if other factors are adjusted, taking care of others (excluding long-term care), having work with income for men, and participating neighborhood associations for women are positive for maintaining good health after 3 years. These results suggest that living with “moderate” responsibilities and roles tends to lead to health maintenance for the elderly, regardless of their home, private, or social environment.

This study included only elderly people in good health in one region of Japan. Therefore, cross-validation in other countries and regions is needed. However, if the results are more generalized, it would be possible to suggest legitimate reasons for the elderly to take on roles at home and in society to help each other.

## Ethics declarations

This study was reviewed and approved by the Ethics Committee, 10.13039/100016213University of Toyama, with the approval number R2021135.

## Data availability statement

Data associated with our study has not been deposited into a publicly available repository. Because the authors do not have permission to share data.

## CRediT authorship contribution statement

**Shota Kuroiwa:** Writing – review & editing, Writing – original draft, Methodology, Formal analysis, Data curation, Conceptualization. **Keiichiro Kita:** Writing – review & editing, Validation, Supervision, Conceptualization. **Maiko Kuroiwa:** Writing – review & editing, Validation, Methodology, Conceptualization. **Shinji Minami:** Writing – review & editing, Validation, Data curation, Conceptualization. **Seiji Yamashiro:** Writing – review & editing, Validation, Supervision, Project administration, Conceptualization.

## Declaration of competing interest

The authors declare that they have no known competing financial interests or personal relationships that could have appeared to influence the work reported in this paper.

## References

[1] Japan Ministry of Health, Labour and Welfare (2019). https://www.mhlw.go.jp/stf/wp/hakusyo/kousei/19/.

[2] Study Group of Experts on Healthy Life Expectancy (2019). https://www.mhlw.go.jp/content/10904750/000495323.pdf.

[3] Kishi R., Horikawa N. (2004). Role of the social support network which influences age of death and physical function of elderly people: study of trends in and outside of Japan and future problems. [Nihon Koshu Eisei Zasshi]. Jpn. J. Publ. Health.

[4] Sato T., Kishi R., Suzukawa A., Horikawa N., Saijo Y., Yoshioka E. (2008). Effects of social relationships on mortality of the elderly: how do the influences change with the passage of time?. Arch. Gerontol. Geriatr..

[5] Holt-Lunstad J., Smith T.B., Layton J.B. (2010). Social relationships and mortality risk: a meta-analytic review. PLoS Med..

[6] Umberson D., Montez J.K. (2010). Social relationships and health: a flashpoint for health policy. J. Health Soc. Behav..

[7] Kuiper J.S., Zuidersma M., Oude Voshaar R.C.O., Zuidema S.U., van den Heuvel E.R., Stolk R.P., Smidt N. (2015). Social relationships and risk of dementia: a systematic review and meta-analysis of longitudinal cohort studies. Ageing Res. Rev..

[8] Kanamori S., Kai Y., Aida J. (2014). Social participation and the prevention of functional disability in older Japanese: the JAGES cohort study. PLoS One.

[9] Fujiwara Y., Amano H., Kumagai S. (2006). Physical and psychological predictors for the onset of certification of long-term care insurance among older adults living independently in a community a 40-month follow-up study. [Nihon Koshu Eisei Zasshi]. Jpn. J. Publ. Health.

[10] Hirai H., Kondo K., Ojima T., Murata C. (2009). Examination of risk factors for onset of certification of long-term care insurance in community-dwelling older people: AGES project 3-year follow-up study. [Nihon Koshu Eisei Zasshi]. Jpn. J. Publ. Health.

[11] Ishikawa Y., Kondo N., Kondo K. (2016). Social participation and mortality: does social position in civic groups matter?. BMC Publ. Health.

[12] Kawachi I., Berkman L.F. (2001). Social ties and mental health. J. Urban Health.

[13] Dahlberg L., McKee K.J., Frank A., Naseer M. (2022). A systematic review of longitudinal risk factors for loneliness in older adults. Aging Ment. Health.

[14] Hilbrand S., Coall D.A., Gerstorf D., Hertwig R. (2017). Caregiving within and beyond the family is associated with lower mortality for the caregiver: a prospective study. Evol. Hum. Behav..

[15] Takeda T., Kondo K., Hirai H., Murata C. (2007). Psychosocial factors as predictors for dementia among community-dwelling older people. Occup. Ther..

[16] Avlund K., Damsgaard M.T., Holstein B.E. (1998). Social relations and mortality. An eleven year follow-up study of 70-year-old men and women in Denmark. Soc. Sci. Med..

[17] Kuroiwa S., Kita K., Kuroiwa M., Yoshida K., Minami S., Yamashiro S. (2021). Do elderly people providing nursing or caring for others help the providers maintain their health? [Nihon Ronen Igakkai Zasshi] Jpn. J. Geriatr..

[18] Natsuhara K. (2018). Daily life support through “mutual aid” in the community-based integrated care system from the perspective of primary health care. Jpn. J. Health Hum. Ecol..

[19] Cabinet Office Policy Commander (Policy Coordination) (2021). https://www8.cao.go.jp/kourei/ishiki/r02/zentai/pdf_index.html.

[20] Steptoe A., Deaton A., Stone A.A. (2015). Subjective wellbeing, health, and ageing. Lancet.

[21] Iwasa H., Kawaai C., Gondo Y., Inagaki H., Suzuki T. (2006). Subjective well-being as a predictor of all-cause mortality among middle-aged and elderly people living in an urban Japanese community: a seven-year prospective cohort study. Geriatr. Gerontol. Int..

[22] Kodama S., Kurimori S., Hoshi T. (2018). Association between feelings of happiness among community-dwelling, independent, elderly individuals in an Okinawan farm village and survival three years later. [Nihon Koshu Eisei Zasshi]. Jpn. J. Publ. Health.

[23] Koyano W. (1987). Measurement of competence in the elderly living at home: development of an index of competence. Jpn. J. Publ. Health.

[24] Chuang M.J. (2017).

[25] Ogawa W., Miyazaki S. (2015). Diagnosis criteria for obesity and obesity disease. Health Eval. Promot..

[26] Haak M., Löfqvist C., Ullén S., Horstmann V., Iwarsson S. (2019). The influence of participation on mortality in very old age among community-living people in Sweden. Aging Clin. Exp. Res..

[27] Murata C., Takaaki K., Hori Y. (2005). Effects of social relationships on mortality among the elderly in a Japanese rural area: an 88-month follow-up study. J. Epidemiol..

[28] Smith S.G., Jackson S.E., Kobayashi L.C., Steptoe A. (2018). Social isolation, health literacy, and mortality risk: findings from the English longitudinal study of ageing. Health Psychol..

[29] Soares M.U., Facchini L.A., Nedel F.B., Wachs L.S., Kessler M., Thumé E. (2021). Social relationships and survival in the older adult cohort. Rev. Latino-Am. Enferm..

[30] Fakoya O.A., McCorry N.K., Donnelly M. (2020). Loneliness and social isolation interventions for older adults: a scoping review of reviews. BMC Publ. Health.

[31] Kuroiwa S., Kita K., Watanabe F. (2016). Do care activities by elderly people lead to an increased sense of purpose in life?. An Official J. Japan Prim. Care Assoc..

[32] Takeuchi R., Kubota A., Takata K., Ohta T. (2013). The relationship between the frequency of physical activity and social capital and changing in quality of life in elderly people—a longitudinal Shizuoka elderly cohort. Jpn. J. Lifelong Sport..

